# Assessment of radio-frequency heating of a parallel transmit coil in a phantom using multi-echo proton resonance frequency shift thermometry

**DOI:** 10.1016/j.mri.2020.12.013

**Published:** 2021-04

**Authors:** Hongbae Jeong, Matthew C. Restivo, Peter Jezzard, Aaron T. Hess

**Affiliations:** a Wellcome Centre for Integrative Neuroimaging, FMRIB Division, Nuffield Department of Clinical Neurosciences, University of Oxford, Oxford, United Kingdom; b Athinoula A. Martinos Center for Biomedical Imaging, Department of Radiology, Massachusetts General Hospital, Boston, MA, USA; c Laboratory of Imaging Technology, Biochemistry and Biophysics Centre, NHLBI, NIH, Bethesda, MD, United States; d Centre for Clinical Magnetic Resonance Research, Department of Cardiovascular Medicine, University of Oxford, Oxford, United Kingdom; e British Heart Foundation Centre for Research Excellence, Oxford, United Kingdom

**Keywords:** RF safety and SAR, MRI temperature measurement, RF transmit coils, Parallel transmission, pTx, 3D, three-dimensional, B_0_, static magnetic field, B_1_, radiofrequency field, B_1_^+^, RF transmit field, BOLD, blood oxygenation level dependent, C_p_, specific heat capacity, CP, circularly polarised, DI, de-ionised, DICOs, directional couplers, EM, electromagnetic, FA, flip angle, me-GRE, multi-echo 3D spoiled gradient echo, PMMA, poly(methyl methacrylate) or acrylic, PRF, proton resonance frequency shift, pTx, parallel transmission, RF, radio-frequency, RMSE, root mean square error, SAR, specific absorption rate, SVD, singular-value decomposition, TIAMO, time interleaved acquisition of modes;

## Abstract

We propose a workflow for validating parallel transmission (pTx) radio-frequency (RF) magnetic field heating patterns using Proton-Resonance Frequency shift (PRF)-based MR thermometry. Electromagnetic (EM) and thermal simulations of a 7 T 8-channel dipole coil were done using commercially available software (Sim4Life) to assess RF heating. The fabrication method for a phantom with electrical properties matched to human tissue is also described, along with methods for its electrical and thermal characterisation. Energy was deposited to specific transmit channels, whilst acquiring 3D PRF data using a pair of interleaved RF shim transmit modes. A multi-echo readout and pre-scan stabilisation protocol were used for increased sensitivity and to correct for measurement-to-measurement instabilities. The electrical properties of the phantom were found to be within 10% of the intended values. Adoption of a 14-min stabilisation scan gave sufficient suppression of any evolving background spatial variation in the B_0_ field to achieve <0.001 °C/mm thermometry drift over 10 min of subsequent scanning. Using two RF shim transmit modes enabled full phantom coverage and combining multiple echo times enabled a 13–54% improvement in the RMSE sensitivity to temperature changes. Combining multiple echoes reduced the peak RMSE by 45% and visually reduced measurement-to-measurement instabilities. A reference fibre optic probe showed temperature deviations from the PRF-estimated temperature to be smaller than 0.5 °C. Given the importance of RF safety in pTx applications, this workflow enables accurate validation of RF heating simulations with minimal additional hardware requirements.

## 1 Introduction

Computational modelling and simulation have long been used for the design, optimisation, efficacy and safety testing of medical devices. Examples include modelling fluid dynamics, electromagnetic (EM) and optical properties, solid mechanics, ultrasound behaviour, and heat transfer [1]. Relevant to magnetic resonance imaging (MRI), EM field simulation is commonly used to estimate RF heating. This is particularly important for static magnetic field (B_0_) strengths of 7 T and above, which are used to provide enhanced signal and contrast for certain applications, such as blood oxygenation level-dependent (BOLD) imaging [2]. Specific absorption rate (SAR) can be substantial since SAR per input power increases with field strength [3,4]. Additionally, the shorter EM wavelength at high field can lead to inhomogeneity in the radio-frequency (B_1_^+^) transmit field. The concept of parallel transmission (pTx) has been introduced to facilitate RF excitation at high field, enabling excitation of complex shapes [5], the lowering of SAR burden via pulse designs with local SAR constraints [6], or for achieving improved B_1_^+^ homogeneity [7].

However, the use of pTx adds complexity to the estimation and monitoring of RF energy deposition [8,9] through difficulties in predicting safe levels of local (10 g) SAR. In particular, when using pTx the local SAR no longer has a simple linear relationship to input energy, but instead has a complex relationship dependent on the superposition of local *E*-fields, which can vary in time during acquisition. Since it is currently not possible to measure thermal elevation in patients during the scan, pre-calculated EM simulation of each transmit channel is required to properly estimate the expected local RF energy deposition [10]. In order to assure patient safety from RF field exposure in MRI these EM simulations in turn require some form of validation. Thermal measurements using a suitable phantom in the scanner can help provide an assurance that the EM simulations are appropriately designed and reflect realistic EM field behaviours [[11], [12], [13], [14], [15], [16], [17]].

Dielectric phantoms are widely used to estimate EM field behaviours and RF heating patterns in MRI. The most common type of phantom is a liquid dielectric phantom fabricated with a combination of sodium chloride (NaCl), sucrose and distilled water [18]. In order to target the dielectric properties of tissue at a specific resonance frequency, the NaCl and sucrose content are adjusted to control the electrical conductivity and permittivity, respectively. Although such phantoms are able to load coils in a realistic way, NaCl-based phantoms are not suitable for MR thermometry experiments. Firstly, excessive use of sucrose can introduce additional spectral components which lead to a complicated signal phase evolution [19]. Secondly, the thermal behaviour is difficult to capture in the liquid state due to high thermal convection within the phantom [20]. As an alternative, polyethene powder has been used to control the permittivity to achieve a phantom suitable for MR thermometry [21] and gelling agents, such as agarose or bovine skin gelatine, can be used to reduce convection by rendering the phantom semi-solid. Additionally, previous studies have shown that agar-gel phantoms are suitable for testing RF heating using an MR thermometry approach and therefore offer the possibility of using such phantoms as a method for validation of EM simulations [11]. Ideally, therefore, it is desirable to design a phantom with well-characterised dielectric properties, along with sufficient convection stability, so that EM simulations can model RF heating in the phantom and these can be validated with experimental measurements.

Care is needed when introducing independent thermal measurement hardware into an MRI environment. In practice, use of fibre optic probes is the most common method to measure absolute temperature changes [22], and these are often used as ground truth in the validation of EM simulations and MR thermometry due to the high accuracy of their thermal measurement [11,23]. The non-magnetic characteristic of fibre optic probes allows location of the probe in the presence of the main magnetic field, but fibre optic probes are only capable of monitoring temperature changes at specific point locations in the sample.

Conversely, MR-based thermometry offers non-invasive temperature measurement throughout the volume of the object under test. For instance, proton-resonance frequency shift (PRF) based thermometry uses the temperature dependence of proton resonance shift (and hence gradient-echo image phase) for thermal estimation [11,24,25]. However, a confound to PRF measurement is the possibility of extraneous influences on the main magnetic field, in particular magnetic field changes arising from gradient coil heating/cooling. Methods to monitor such unwanted field drifts include using oil-filled samples as fiducial markers that have a negligible thermal PRF response [11,26]. Also, hardware solutions such as using B_0_ field cameras to monitor extraneous field variations, or the use of several thermal probes (4 or more) to capture the spatial gradients of absolute temperature changes, have been introduced [[27], [28], [29]]. A drawback of these approaches is that hardware for monitoring such magnetic field changes may not be readily available to many research groups, and the use of multiple thermal probes can add complexity to phantom design and EM simulations.

In this study, we (i) propose a method for design of a phantom and measurement of its dielectric and thermal properties, and (ii) assess the impact of electrical and thermal phantom properties on induced RF heating via EM simulations. Finally, we (iii) introduce a multi-echo PRF technique to assess heating of the phantom in a 7 T pTx coil. Multiple echo times are adopted for increased accuracy and to reduce measurement-to-measurement instability, along with a time interleaved acquisition of modes (TIAMO) to reduce B_1_^+^ dropout. To address gradient heating-induced field changes, a stabilisation pre-scan method is evaluated. The RF heating generated from the pTx RF transmit coil is assessed both in simulation and experiment, and error sensitivity analyses of electrical and thermal properties are compared.

## 2 Methods

### 2.1 Phantom preparation and characterisation

**Fig. 1:**
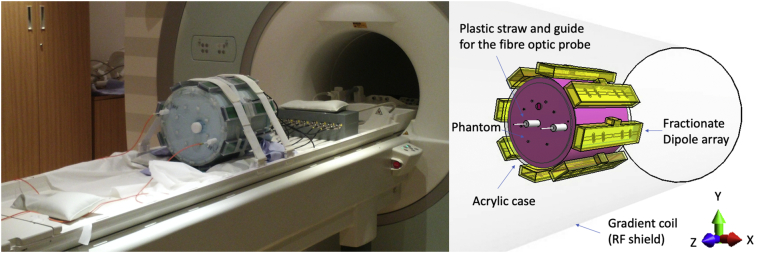
Experimental set-up. showing the phantom, coil, and fibre optic probes (left) and the model of the 8-element dipole array inside a gradient coil acting as an RF shield (right).

A cylindrical acrylic (PMMA) container was fabricated with a diameter of 280 mm, 300 mm height and 5 mm thickness (Advanced Fabrications Ltd., London, UK). Several 3 mm diameter holes were made in the endplate to guide fibre optic probes into the centre of the phantom using plastic straws and plastic guides on top of the phantom (Fig. 1). The phantom recipe contained the following proportions per litre of de-ionised (DI) water: agar (20 g, Sigma-Aldrich, Natick, MA, USA), polyethylene powder (150 g, Sigma-Aldrich, Natick, MA, USA), sodium chloride (4 g, Sigma-Aldrich, Natick, MA, USA), TX-151 (68 g, Oil Center Research International, Lafayette, LA, USA), and benzoic acid (30 g, Fisher Scientific UK Ltd., United Kingdom), with a total phantom volume of 16.8 l [21] (note that sodium azide in the cited recipe was replaced with benzoic acid - a common preservative used in the food industry - to reduce the chemical safety risk). This recipe targeted the average electrical properties of brain white matter and grey matter at 297.2 MHz (ε_r_: 51; σ: 0.57 S/m). The setting compound TX-151 was included to reduce the setting time [21].

In order to reproduce the properties of the phantom in silico*,* the dielectric and thermal properties (specific heat capacity, thermal diffusivity, thermal conductivity, and heat transfer coefficient) were characterised independently. In the case of the electrical parameters, the dielectric properties of the phantom were measured with an open-ended coaxial probe and network analyser, and saline water was used for verification of the measurement [30]. The specific heat capacity of the phantom was estimated using the weighted mass and specific heat capacity of each component in kcal/kg/°C [TX-150 (equivalent to TX-151): 0.300; polyethylene powder: 0.520; H_2_O: 1.000; NaCl: 0.217; Benzoic acid: 0.288; Agar: 0.382] [[31], [32], [33]]. Thermal diffusivity of the phantom, K, was measured using a laser flash system LFA 427 (NETZSCH-Gerätebau GmbH, Germany), averaged five times across four different samples (K = 0.126 ± 0.00054 cm^2^/s). This value is then used to calculate thermal conductivity (*κ*), via measurements of density (ρ) and specific heat capacity (C_p_) using Eq. (1).

(1) $$\kappa =\frac{K}{\rho ∙C_{p}}$$

**Fig. 2:**
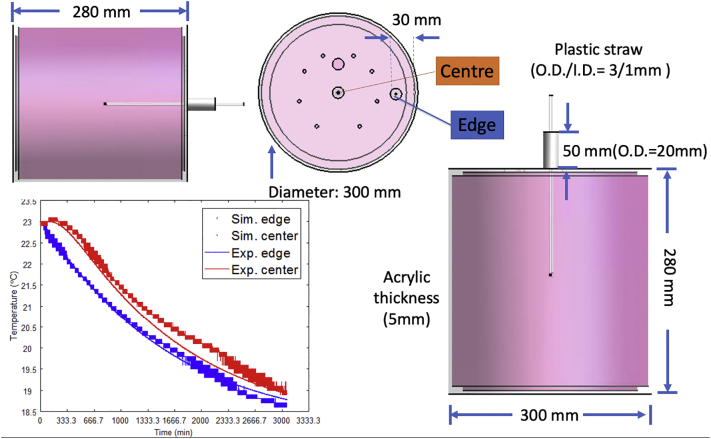
Results from the phantom cooling experiment to determine the heat transfer coefficient (thermal coupling of the phantom to its environment) and the same from the Sim4Life thermal simulation (left-bottom), agar-gel phantom drawing in Sim4Life (right).

The heat transfer coefficient determines the thermal coupling of the phantom to its environment. The heat transfer coefficient was calculated by storing the phantom in a room controlled to 23 °C for two days and then subsequently placing the phantom in the scanner room, which was maintained at a controlled temperature in the range of 18.4 to 18.6 °C measured with a fibre optic probe (Note that the absolute temperature of the phantom was measured using fibre optic probes with field dependency [22]. This contains some error as the absolute temperature was measured on the patient table outside of the bore which was not 0 T). The temperature of the phantom was then monitored using a 2-channel fibre optic probe (Neoptix, Québec City, Canada) for 47 h, one probe placed at the edge of the phantom and the other at the centre. The observed temperature changes were then compared to matched simulations in Sim4Life with varying heat transfer coefficients, assessed between 1 W/m^2^/°C and 10 W/m^2^/°C with a step size of 0.5 W/m^2^/°C (Fig. 2).

### 2.2 Pulse sequence to evaluate pTx RF heating using proton resonance frequency shift

**Fig. 3:**
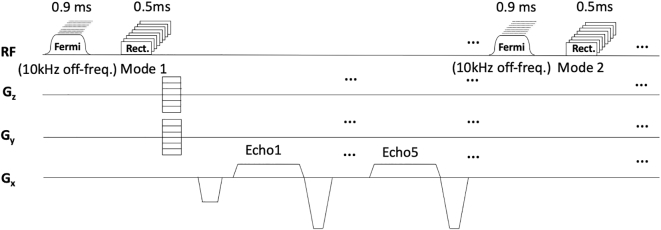
3D GRE sequence diagram for PRF MR thermometry. Each heating experiment was performed by applying a 0.9 ms Fermi RF heating pulse at 10 kHz off resonance on the chosen transmit channel prior to all excitation pulses. Multi-echo GRE was set to acquire images in two transmit configurations.

A 7 T Magnetom human MRI scanner (Siemens Healthineers, Erlangen, Germany) was used in conjunction with an 8 channel transmit/receive dipole coil array [34]. A pulse sequence was programmed to deposit RF energy on a specified channel and measure the resultant heating using all channels with PRF thermometry. For this purpose, a multi-echo 3D spoiled gradient echo (me-GRE) was adapted by adding a channel-specific, 10 kHz off-resonance Fermi RF pulse, with a duration of 0.9 ms, prior to the RF image excitation and calibrated to deposit 5.50 W average power to the coil plug (additional information and code describing the transmitted power calculation is provided in Supplemental Information Section 4). In order to achieve adequate volume coverage, the me-GRE readout was set to acquire images in two transmit configurations, in an interleaved manner according to the TIAMO [35] regime. Fig. 3 shows the pulse sequence diagram of the PRF sequence. The image matrix size was set to 64 × 64 × 30, with a field of view of 300 × 300 × 300 mm^3^, and an elliptical k-space coverage. The protocol resolution was 4.7 × 4.7 × 10 mm^3^, TR = 14 ms, and TEs of 1.44 ms, 3.62 ms, 5.5 ms, 7.38 ms and 9.26 ms, excitation pulse voltage: 27.78 V (flip angle: 10°), and temporal resolution: 41 s. Thirty image repetitions were acquired (total heating duration 21 min) after stabilisation of gradient-induced heating. The imaging excitation pulse contributed 0.55 W to the deposited RF energy per channel.

To evaluate the B_0_ stability a one hour scan (86 measurements) without heating was acquired using the same sequence used in the heating experiment while monitoring the absolute temperature using a two-channel fibre optic probe. The PRF heating maps were reconstructed as below, and first-order (X, Y, and Z) temperature gradients and bulk (spatially invariant) drift in temperature were calculated at each time point. The results from this stabilisation experiment were used to determine the number of heating-free scans necessary to establish thermal measurement stability (i.e., no remaining artefactual spatially evolving temperature distribution) before the main heating experiment could be performed.

To calculate the PRF-induced temperature shift the images were first reconstructed by applying a Hann window filter in all three dimensions and zero padding k-space to 128 × 128 × 60. Two different reconstructions were carried out. The first reconstruction used only the first image acquired with 45° phase shift between transmit channels (TIAMO mode 1). The second reconstruction used both the mode1 image and a second image which had 0° phase shift between transmit channels (TIAMO mode 2), reconstructed by concatenating them in the receive dimension, giving 2 × 8 receive coil images which were combined using singular-value decomposition (SVD) using all the time points and echoes [36]. The first echo of each measurement (TE = 1.44 ms) was used to phase all subsequent echoes; this is expected to remove measurement-to-measurement instabilities of the transmit/receive chain. According to the results of the B_0_ drift stabilisation experiment (see Section 3.2) the 20th repetition of the stabilisation scan was used as a phase reference, and temperature maps were calculated using Eq. (2):

(2) $$∆T=\frac{\Delta \phi -{\Delta \phi }_{\mathrm{drift}}}{\gamma \alpha B_{0}\mathrm{TE}}$$

Where γ = 42.576 MHz/T, α = −0.00988 ± 0.0065 ppm/°C [[37], [38], [39]], B_0_ = 7 T, TE = echo time and Δϕ_drift_ is derived from the two-channel fibre optic probe at the centre and edge of the phantom [24,37]. The remaining system frequency drift was corrected using the field drift information recorded during a non-RF heating experiment, and the correction results were rechecked against the measured temperature from the two-channel fibre optic probes. The temperature maps from the second to fifth echoes were combined by weighting them according to the product of their respective echo times and image intensities.

B_1_^+^ maps in units of Hz/V were measured using a pre-saturation B_1_ mapping method (Siemens-WIP543, TR/TE: 10 s / 1.97 ms) which acquires two turbo-flash images where the second image has a channel-specific non-selective saturation RF pulse applied prior to the readout train. To calibrate heating in absolute units the B_1_^+^ maps were calibrated according to the voltage delivered to the coil. Also, cable losses (including those within the T/R switch) between coil plug and coil port were measured with a network analyser on the bench as −1 dB, and the reflected voltage from mismatch between the power amplifier and coil plug was measured using the scanner's DICOs using a channel-multiplexed measurement, as in reference [40]. Two fibre optic probes were positioned, one in the centre of the phantom, and another 30 mm from the inner edge of the phantom. The 8-channel dipole array was placed equally around the phantom as shown in Fig. 1. After heating experiments on each element, the phantom was left to cool until reaching equilibrium temperature before testing the neighbouring element (3 h of cooling time).

### 2.3 Electromagnetic and thermal simulation of RF coil and phantom

All simulations were performed using Sim4Life (ZMT, Zürich, Switzerland). The set-up included a model of the 8-channel transmit/receive dipole array (MR Coils BV, Zaltbommel, The Netherlands) [34] placed symmetrically around the cylindrical phantom shown in Fig. 2. The coils do not have tuning capacitors, and the matching capacitor values were chosen by selecting values that gave in simulation the same reflection coefficient for each port in the dipole array as those measured using the scanner directional couplers (DICOs) under experimental conditions. A model of the 7 T scanner's SC 72 gradient coil (Siemens Healthineers, Erlangen, Germany) is included as an RF shield to account for reflected radiation from the RF shield (Length: 158 cm, Diameter: 64 cm). This was simulated as a surface with perfect electric conductivity (PEC) properties. The electrical and thermal properties of the phantom contents were set to the values measured using the protocol described in Section 2.1 and the shell of the phantom (5 mm thick PMMA) was set to ε_r_: 3.2, σ: 10^−9^ S/m. The grid resolution was set to 1 × 1 × 1 mm^3^ for the RF coil and the phantom. The estimated B_1_^+^ fields were extracted from the EM simulation and normalised to an input voltage of 1 V to compare with the experimental results. The EM simulations were normalised to an averaged transmit power of 5.50 W, as generated in the PRF thermometry experiments (i.e., the value ultimately achieved at the coil location in practice, see Supplemental Material Section 4 for details of the calculation method used) and subsequently used as inputs to the package's Pennes' bio-heat equation provided in Sim4Life. The transient thermal solver was used with the thermal properties of the phantom (characterised in Section 2.1). A mixed boundary condition was chosen with the stabilised phantom, using a background temperature of 18.6 °C, heat transfer coefficient of 2.5 W/m^2^/°C, and a simulation that ran for 21 min duration. The heating maps calculated in the plastic container (outer 5 mm layer) were excluded in post-processing as this region is not visible in PRF thermometry. A point simulation sensor was placed for thermal estimation at the same location as the position of the fibre optic probe. The probe was localised in the experimental MRI data by tracing the signal void generated by the plastic straw and the depth determined as the length of insertion of the fibre optic probe.

### 2.4 Sensitivity analysis of the EM simulation

The methods used to conduct the sensitivity analyses were based on the work of Neufeld et al. [41]*.* A sensitivity analysis of the thermal and EM simulations was performed by running a series of simulations in which different values of each parameter were set across 10% changes in phantom parameters and a realistic range that could occur during the experiment to gauge their impact on the thermal simulation results. The default simulation values were based on the results of phantom characterisation and the RF heating experiments described in 2.1, 2.2 (e.g., duration of heating: 1258 s). The parameters considered were those that could occur due to either measurement inaccuracy (phantom dimension, reflection coefficient, electrical conductivity, relative permittivity, density, specific heat capacity, thermal conductivity, and the heat transfer coefficient) or experimental design (probe location, MRI bore temperature, RF exposure time). The sensitivity factor was calculated by assessing percent temperature changes relative to a percent change of the default value (i.e., S/m, kg/m^3^, etc.). The measurement standard deviation of each parameter, given as a percentage of the default parameter value, represents the accuracy with which the parameter is known, these were derived from the literature [41]. Uncertainty was calculated by multiplying the sensitivity factor with the standard deviation.

## 3 Results

### 3.1 Phantom preparation and characterisation

The estimated and measured electrical properties of the phantom are listed in Table 1. For verification of the electrical measurements, the dielectric properties of 51.3 mM saline water were also measured, showing a measurement accuracy of within 1.6%. The dielectric properties of the phantom were measured as ε_r_ = 47.76 and σ = 0.62 S/m for relative permittivity and conductivity, respectively, and these measured values were used in the EM simulation. Agreement to within 10% was found between the target and measured values for relative permittivity and conductivity (Table 1). Fig. 2 shows the measured temperature changes from the phantom cooling experiment (solid line) and the results of thermal simulation (dotted line). From these data, the heat transfer coefficient between the surface of the phantom and the surrounding air was estimated as 2.5 W/m^2^/K and is the value used for thermal estimation in the subsequent EM and thermal simulations.

**Table 1:** Measured dielectric properties. The method used was based on the work of Zajíček et al. [30]. 51.3 mM Saline water was used to verify the accuracy of the measurement, and the fabricated phantom properties were measured using an open-ended probe.

Material	51.3 mM Saline		Phantom	
	ε_r_	σ (S/m)	ε_r_	σ (S/m)
Estimated value	77.98	0.60	51.00	0.57
Measured value	77.62	0.59	47.76	0.62
Difference	0.4%	1.6%	−6.4%	+8.7%

### 3.2 Pulse sequence to evaluate pTx RF heating using proton resonance frequency shift

**Fig. 4:**
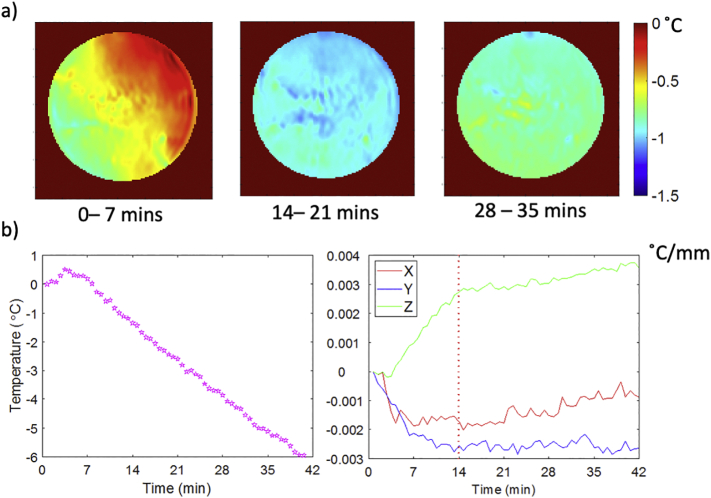
For the case of the no-RF heating experiment: a) shows temperature drift maps over various 7-min intervals: 0–7 min, 14–21 min, and 28–35 min. b) shows the average (spatially independent) apparent temperature drift over the phantom (left graph) and 1st order fitting in X, Y, and Z of the variation in apparent temperature drift over repetitions (right graph).

The spatial gradient of apparent temperature change due to B_0_ drift is plotted against scan duration for the no-RF-heating stability evaluation scan in Fig. 4. The spatial gradients in the temperature maps stop evolving after 14 min of scanning. However, the average (spatially independent) temperature offset continues to increase throughout the scan. A stabilisation period of 20 repetitions (= 13 min, 58 s) with a 0 V heating pulse on all channels was determined as a sufficient period to achieve negligible remaining spatially-dependent gradient heating effects, based on this calibration scan.

Fig. 4, Fig. 6 shows the temporal standard deviation of the PRF temperature measurements in a central slice over the latter period of the no-RF-heating scan, shown calculated with and without combination of the echo times, and transmit states. The average and peak standard deviation in apparent temperature change across the phantom reduced from 0.062 to 0.044, and from 6.960 to 0.302 °C, respectively, by combining the two transmit states. The average standard deviation reduced from 0.078 to 0.050 °C for echoes 2 to 5, and a marginal improvement was found when combining the 4 echoes. The peak standard deviation reduced from 0.289 to 0.188 °C for echoes 2 to 5 and was further lowered to 0.160 °C when combining the 4 echoes.

**Fig. 5:**
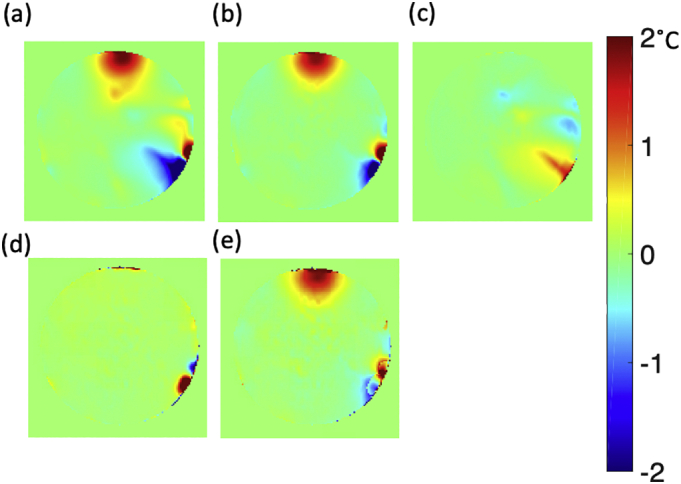
Combined echo evaluation of the effectiveness of phasing the data with the first echo which mitigates the non-RF related artefact. a) PRF reconstruction affected by artefact without TE1 artefact mitigation, b) PRF reconstruction after the TE1 artefact mitigation which is the non-RF heating related artefacts, c) Difference in temperature between PRF reconstruction without TE1 artefact mitigation and with TE1 artefact mitigation, d) Calibrated from the stabilisation period, e) Artefact mitigation using calibrated artefact from stabilisation period. (The artefact shown in PRF reconstruction is not an RF heating related artefact, and is potentially caused by the laminated print label attached outside of the phantom (see Supplemental Fig. 3).

**Fig. 6:**
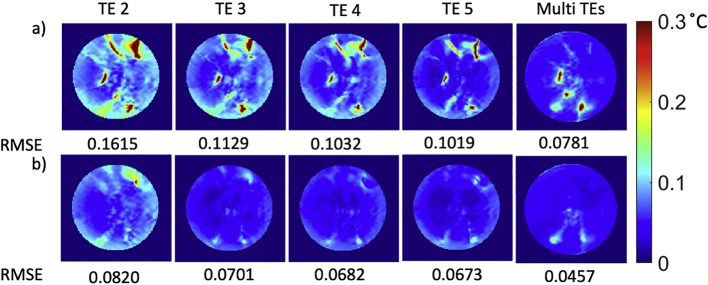
Standard deviation of PRF difference map in degree Celsius over each repetition in the central slice of the phantom (The data were acquired without external RF heating with a fermi pulse). a) shows the results of a single shim (mode1) acquisition, b) shows results of a 2-RF-shim TIAMO acquisition (mode1 an mode2).

The effectiveness of phasing the data with the first echo is compared in Fig. 5. Fig. 6 compares the RMSE when using a single echo to averaging all echoes and the effect of using transmit TIAMO mode1 (a) or TIAMO mode 2 (b).

### 3.3 Sensitivity and uncertainty analysis of the EM simulation

The sensitivity of the simulation to uncertainty in various material properties is compared in Table 2, showing temperature changes at the edge of the phantom after 21 min of the heating. It is evident from Table 2 that the permittivity, density and specific heat capacity of the phantom are the three most sensitive parameters to characterise accurately in phantom preparation. The total uncertainties from phantom characterisation are assessed as 7.78%. The largest overall error that could be caused by experimental control is likely to be the x position of the fibre optic probe, showing a 9.21% uncertainty in temperature elevation for a realistically possible error in probe position characterisation. Other than the position of the probe, the uncertainties of RF exposure time is shown as 1.52%. The total uncertainties caused by parameters under experimental control is calculated as 17.94%.

**Table 2:** Input parameter sensitivity analysis. The Default Values are the values used for the main simulations, whereas the Adjusted Values were set to realistic values that could occur due to either measurement inaccuracy or experimental design choice. The sensitivity factors were calculated as the percentage error in temperature change per percentage error of input parameter. The measurement standard deviation (% of default value) was derived from literature values [41]. *Phantom was thermally stabilised for two days in the MRI scanner room prior to the experiment. Thus, the initial temperature of the phantom and MRI bore are assumed to be the same (Note that the absolute temperature was measured using fibre optic probes with field dependency [22]. This contains some error as the absolute temperature was measured on the patient table outside of the bore where the field strength is not 0 T). **The positioning error of the fibre optic probe was chosen as the half of the PRF image resolution used in the experiment. ***The standard deviation of the alpha coefficient was derived from three literature studies involving 1–2% agar phantoms [[37], [38], [39]].

Parameter	Default Value	Adjusted Value	Thermal Elevation using Default Value [°C]	Thermal Elevation using Adjusted Value [°C]	Sensitivity Factor	Measurement Std. Dev. (% of default value) [41]	Uncertainty [%]
Simulation parameters driven from Phantom Characterisation							
Phantom conductivity [S/m]	0.6227	0.6850	0.8643	0.8805	0.19%/%	6.42% (0.04 S/m)	1.20%
Phantom permittivity	47.76	52.54	0.8643	0.8662	0.02%/%	5.86% (2.8)	1.29%
Phantom density [kg/m^3^]	1022.22	1124.40	0.8643	0.7882	0.88%/%	0.98% (10 kg/m^3^)	0.86%
Phantom heat capacity [J/kg/°C]	3776.3	4153.9	0.8643	0.7882	0.88%/%	4.24% (160 J/kg/°C)	3.73%
Phantom thermal conductivity [W/m/°C]	0.4864	0.5350	0.8643	0.8618	0. 03%/%	4.11% (0.02 W/m/°C)	0.12%
Phantom heat transfer coefficient (W/°C/m^2^)	2.5	3.0	0.8643	0.8642	0.01%/%	40.00% (1 W/°C/m^2^)	0.58%
Total uncertainties in simulation from Phantom characterisation							7.78%

Simulation parameters driven from Experiment (PRF thermometry and fibre optic probe)							
Stabilised temperature of the phantom and background* [°C]	18.6	22	0.8643	0.8643	0.0%/%	9.30% (1.73 °C)	0.00%
Measured magnitude of S_ii_ [S_11_ in dB]	0.4124 [−7.69 dB]	0.3928 [−8.11 dB]	0.8643	0.8231	1.06%/%	4.75% (0.42 dB)	5.03%
RF exposure time [s]	1258	1263	0.8643	0.8675	0.93%/%	1.63% (20.5 s)	1.52%
Exp. Error in fibre optic probe X position [mm]	0	2.5	0.8643	0.9377	3.40%/mm	2.71 mm **	9.21%
Exp. Error in fibre optic probe Y position [mm]	0	2.5	0.8643	0.8515	0.59%/mm	2.71 mm **	1.61%
Exp. Error in fibre optic probe Z position [mm]	0	5	0.8643	0.8686	0.10%/mm	5.77 mm **	0.57%
Total uncertainties in simulation from experiment (PRF thermometry and fibre optic probe)							17.94%

Parameter used for PRF thermometry calculation							
PRF alpha coefficient [ppm/°C]	0.00988	0.01000	0.9491	0.9401	0.14%/%	6.88% (0.00068***)	0.95%

### 3.4 Electromagnetic and thermal simulation of RF coil and phantom compared to measurements

**Fig. 7:**
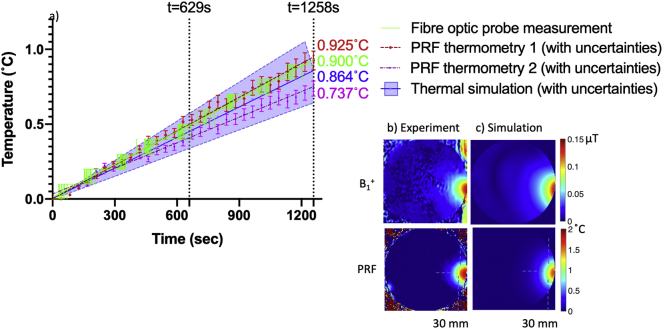
Results showing the B_1_^+^ transmit field and temperature map when heating is applied via coil Channel 2. a) shows the relative temperature changes at the edge of the phantom (30 mm inside), as measured by the fibre optic probe, versus simulation and PRF (using a 4 voxel ROI); uncertainties caused by the PRF alpha coefficient are displayed with error bars, and the total uncertainties in the simulation analysed in Table 2 are also highlighted in purple. Temperature measurements using fibre optic probes from different experimental dates on under identical conditions are plotted to indicate a potential error for the period between 600 s and 900 s. b) shows the measured B_1_^+^ field and PRF, and c) shows the results of their respective simulation. (For interpretation of the references to colour in this figure legend, the reader is referred to the web version of this article.)

The results of the EM and thermal simulations when heating is applied via Channel 2 of the coil are compared in Fig. 7 versus experimental thermometry measurement using PRF MR and fibre optic probe thermometry. The estimated B_1_^+^ field pattern is compared with the measured B_1_^+^ field (top row), and the estimated RF heating profile is compared with measured RF heating profile from PRF thermometry (bottom row). Fig. 7a shows the relative temperature changes at the edge of the phantom, as measured by the averaged fibre optic probe (green), simulation (blue), and PRF (red). The maximum error is found to be between 0.025 and 0.16 °C at the end of the PRF scan (*t* = 21 min, measured twice) when compared with the measurement of the point sample temperature using the fibre optic probe.

**Fig. 8:**
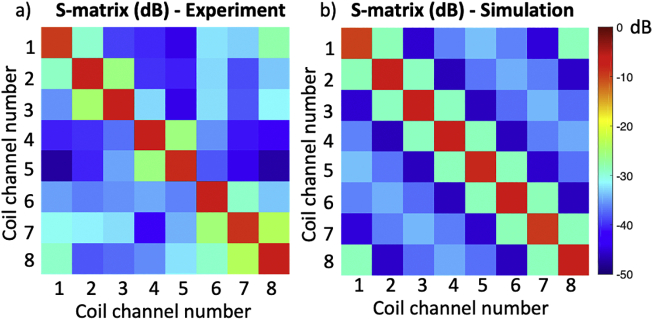
Measured S-parameters in dB (left), compared with the simulated S-parameters in dB (right).

**Fig. 9:**
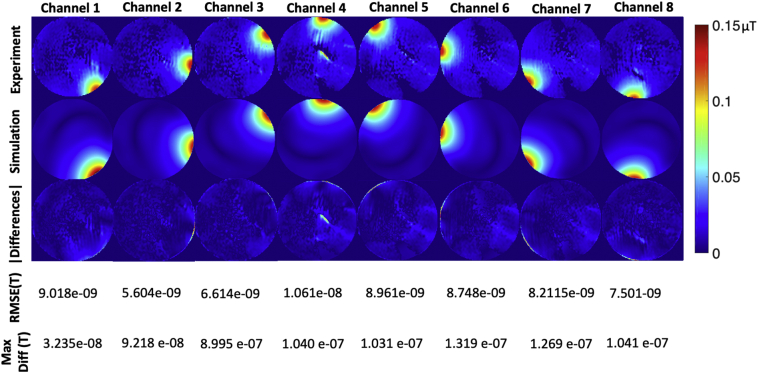
Individual B_1_^+^ transmit fields measured for each channel (top). Simulated B_1_^+^ transmit fields (middle), and difference in B_1_^+^ field between measured and simulated results with RMSE (bottom).

**Fig. 10:**
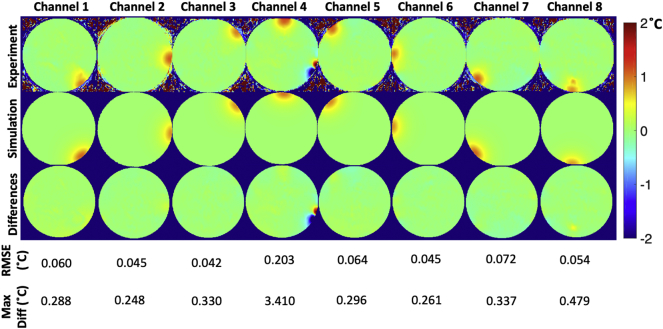
Temperature mapping at the mid-point of the heating duration (*t* = 629 s). Measured PRF thermometry in each channel for the central slice (top), simulated thermal elevation in each channel for the central slice (middle), and differences in °C between measured and simulated thermal elevation (bottom) with RMSE.

**Fig. 11:**
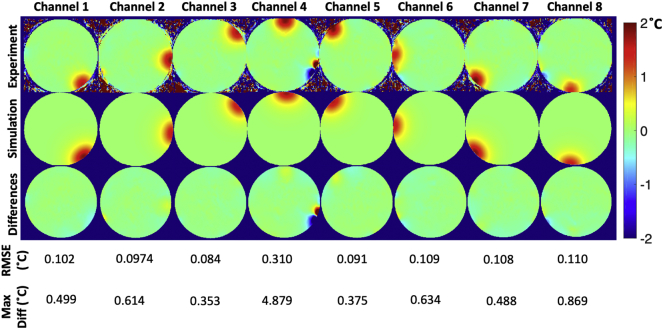
Temperature mapping at the end of the heating duration (*t* = 1258s). Measured PRF thermometry in each channel for the central slice (top), simulated thermal elevation in each channel for the central slice (middle), and differences in °C between measured and simulated thermal elevation (bottom) with RMSE.

Fig. 8 shows the measured S-parameters in dB, compared with the simulated S-parameters in dB. Fig. 9 shows the measured B_1_^+^ maps compared with simulated B_1_^+^ maps for all channels, and Fig. 10 and Fig. 11 show the measured PRF temperature maps compared with the simulated thermal elevation with root-mean-square error (RMSE) at the mid-point of the heating duration (*t* = 629 s) and at the end of the heating duration (*t* = 1258s) when heating each of the 8 channels in turn. The maximum difference in B_1_^+^ field between experiment and simulation was found to be 8.995 e-07 T, and the maximum temperature error between simulation and experiment results was found to be 4.879 °C near the non-RF related artefact.

## 4 Discussion

In this study, we have proposed a procedure for RF safety phantom characterisation, and a pulse sequence to measure channel-specific RF-induced heating using PRF thermometry.

### 4.1 Phantom preparation and characterisation

In the proposed method, TX-151 was used to accelerate the phantom setting time. For the size of phantom proposed here, more than 6 h of setting time was needed to cool down the solution to below 39 °C, whereas with TX-151 the setting time was reduced to a few minutes [21]. A long setting time causes loss of water from the target recipe and requires continuous stirring to spread the polyethylene powder uniformly. Alternatively, polyvinylpyrrolidone can be used to control the permittivity, which is free from sucrose and water-soluble polymer [19]. Care is needed for storage of agar-gel based phantoms, since water evaporation was observed over time [21]. Despite sealing of the phantom lid with silicone, water evaporation was observed over 6 months storage of the phantom in the MR scanner room. As such, the density of the phantom should be recalculated periodically using updated weight and volume measurements for simulation parameters.

In previous studies, TmDOTMA^−^ has shown capability for MR thermometry with enhanced chemical shift temperature sensitivity (0.7 ppm/°C) compared to protons (0.01 ppm/°C), and which could, therefore, be considered for use in a MR thermometry phantom [[42], [43], [44]]. We chose not to use this chemical, however, due to its high cost for a large-volume phantom and the experimental complexity introduced into acquisition of the spectrum. Nevertheless, one could consider TmDOTMA^−^ as a more thermally sensitive chemical to improve MR thermometry sensitivity.

### 4.2 Pulse sequence to evaluate pTx RF heating using proton resonance frequency shift

Oh et al. have successfully shown the capability of MR thermometry both in phantoms and for in-vivo scans of the extremities using a GRE sequence at 3 T [11]. The proposed study is not intended for in-vivo temperature mapping, thus a fast acquisition method did not need to be implemented. For cases where a 3D GRE sequence would be too time-consuming, Echo Planar Imaging (EPI) sequences can be used [45]. An alternative method of in-vivo MR thermometry uses compressed sensing [46]*.* A reduced scan time can be achieved via use of abundant data points that are free from RF heating. In addition, Winter et al. have demonstrated rapid MR thermometry in RF hyperthermia using shortening of the T1 relaxation time [47]. Fast sequences also allow monitoring of temperature in an MR environment for techniques such as RF ablation [48].

The importance of the pre-heating stabilisation scans is shown in Fig. 4, which minimised any evolving B_0_ field gradient drifts without requiring additional hardware. In our system, spatial field variation was stabilised and reached a steady state in 14 min. The time requirements for stabilising the gradient coil may be different on other scanners, as they will depend on the amount of passive shim steel used during magnet installation, so phase drift calibration should be done for each scanner and PRF protocol individually. The advantage of mitigating field drifts in this way is that additional oil compartments or arrays of temperature probes do not need to be used for PRF map correction, although a single (spatially invariant) drift correction may still need to be made, as was the case for our system. Despite this, a residual drift still remained in our data (some of them might be the residual temperature from the previous heating experiment), which resulted in a slight apparent temperature decrease in remote areas of the phantom. The drift error can be mitigated with an additional reference measurement with oil compartments or arrays of fibre optic probes.

It is desirable to induce heating with a different transmit configuration to that used for imaging and enable flexibility in evaluating heating of different pTx configurations or channels. By applying RF heating on one channel at a time the magnitude of E-fields are validated, however this is insufficient to validate a dynamic local SAR model which additionally requires validation of heating with all channels transmitting together. Our proposed PRF measurement sequence simply uses two transmit states. This simple method was applied to avoid the need to use more elaborate pTx sequences, such as spokes RF pulses or universal pulses [49], etc., which provide enhanced coverage of the phantom by reducing error in regions where otherwise there would have been a B_1_^+^ void (See Supplemental Fig. 5). By using a weighted average of multiple TEs a reduction in the variance of the calculated temperature was found, compared to using a single echo time, particularly in regions of lower signal. Higher accuracy means potentially less time needed to measure heating, which improves the accuracy of thermometry.

Furthermore, phasing the multi-echo measurements using the first echo removed some artefactual temperature changes remote from the heating location and provided smoother heating profiles. Such phase changes could originate from changes in transmit or receive paths, for example where a single pTx amplifier drifts slightly, causing a small phase change in regions affected by its transmit sensitivity and that of other channels. Although this method has removed some of the artefact, a residual amount remains (Fig. 5). The residual artefact was dominant near the position of the laminated labels attached to the phantom acrylic which was an institutional safety requirement (Supplemental Fig. 3). In case of phantom labelling, the position and material of any printed label should be chosen carefully to avoid a source of artefact in PRF thermometry. There was also a small and occasional artefact shown at the bottom left of the phantom (Fig. 10, Fig. 11). This residual artefact could be explained by a change in a susceptibility boundary at the edge of the phantom, likely caused by inadequately dissolved phantom ingredients that were occasionally included in the field-of-view (Supplemental Fig. 3).

### 4.3 Electromagnetic and thermal simulation of RF coil and phantom

Simulating an EM field in software allows many parameters to be configured, including geometric properties, coil capacitor values and simulation spatial resolution, all of which can alter the results of the simulation [[50], [51], [52]]. In the case of pTx, any deviation of the simulation from reality is compounded by the interaction of multiple channels (which is hard to adequately model in simulations) and injects uncertainty into SAR prediction, which can require the adoption of prohibitively restrictive limits in order to ensure safety. To gain confidence in the validity of such simulations, measurement of the B_1_^+^ or *E*-fields is required [8]. B_1_^+^ fields can be measured trivially via MRI techniques, however E-fields cannot be measured with standard MR equipment. An alternative is to map the thermal effect (heating) of these fields on the sample as a surrogate (with temperature ultimately, in fact, the safety parameter of most direct safety relevance). Although impractical to achieve in the case of in-vivo imaging, heating effects can be measured in representative phantoms for each element of a transmit array and for different transmit configurations.

### 4.4 Sensitivity and uncertainty analysis of the EM simulation and experiment

For a simplified model system (e.g., a single surface coil element and a homogeneous phantom), less than 1% error in temperature for simulations versus PRF experiments has been reported [11]. In the case of pTx coils, temperature discrepancy between experiments and simulations has been reported as less than 18% using a homogeneous phantom measured with PRF thermometry [8]. This shows that discrepancies can be elevated when neighbouring coil elements are present, due to the complex interaction between coil elements which is not trivial to estimate. A dipole transmit array has a relatively simple geometry compared to other pTx coils. Nevertheless, a dense grid setting (e.g., 0.8 mm × 0.8 mm × 0.8 mm) was necessary to account with sufficient accuracy for the curvature of the meander structures in the FDTD solver, and arbitrary placement of multiple coils required a large amount of computational graphic memory. More efficient graphic memory usage can be achieved by use of localised dense gridding via the subgrid package available in Sim4Life. Also, an accurate description of the fractionated dipole array for 7 T MRI is openly available to the MRI community via the generosity of the group at UMC Utrecht that allows for arbitrary accuracy in simulation [53]. Given the fact that the fractionated dipole array uses only two capacitors at the matching port of each element, this reduced the amount of effort required to optimise the coil in simulation [54]. However, more consideration would be needed for other coil arrays, such as matching the in-situ complex S-parameter values, decoupling circuitry, and capacitor values used in reality [55,56]. It is reported that the agreement between experiment and simulation in temperature measurement is relatively high when the duration of the heating is short (e.g., 132 s for the study by Hoffmann et al. [8], 120 s for the study by Oh et al. [11]). In accordance with these reports, we also observed a good agreement when the duration of heating was less than 13 min (Fig. 7). Unlike previous approaches, we extend our measurement up to 21 min. The primary reason for this long duration was to protect the physical coil elements from excessive input current within a short time duration, but the accuracy of the PRF is reduced after 13 min of heating. This effect could be explained by the uncertainty in the simulated electrical and thermal properties of the phantom, as these uncertainties accumulate error with increasing simulation time.

Characterising the thermal conductivity and heat transfer coefficient was shown in our heating experiment to be not as critical as might be expected since the heating duration is short and the induced temperature rise is small. The sensitivity factor in these parameters is below 0.01% per % change of each parameter, resulting in a change in the predicted temperature rise of less than 0.3% in the simulation (Table 2). The uncertainty analysis was calculated for depositing energy over a 1258 s duration, with a longer duration of energy deposition resulting in greater uncertainty from thermal properties. The total uncertainty derived from phantom characterisation was 7.78%. Thus, care is needed to measure the mass density, permittivity and specific heat capacity of the phantom as well as ensure a reliable sensor location. Regarding uncertainties that may arise from factors under experimental control, the x- and, y- position of fibre optic probe showed the greatest uncertainties in thermal simulation, which are 9.21% and 1.61%, respectively. To increase position accuracy, a higher resolution PRF image may be required to minimise the error. The RF exposure time gives rise to up to 1.52% added uncertainty that is limited by the temporal resolution of the PRF sequence, and the compound uncertainty from experimental control is assessed as 17.94%. The experimental source of error returned three times higher uncertainties than phantom characterisation for thermal simulation, which emphasises that experimental design and control is a critical factor to minimise the error in thermal simulation as well as accurate phantom characterisation. This method of uncertainty analysis assumes all parameters are independent, however covariance may exist between parameters, for example sensor position will interact with thermal diffusivity, and heat capacity with time.

There are also uncertainties in experimental measurement. We have chosen an averaged alpha coefficient from three well-designed studies on PRF alpha coefficient calibration measurement conducted on 1–2% agar gel phantoms (whose tolerance was shown in Fig. 7). [[37], [38], [39]]. The changes were marginal within the given RF heating condition, but the alpha coefficient must be carefully considered for the studies related to high-thermal rise, such as hyperthermia or implant safety testing.

Fibre optic probes give a ground truth temperature elevation, thus essential for RF heating studies. The resolution of the temperature measurement for our fibre optic probes was 0.1 °C that could induce inaccuracy in the temperature measurement up to ±0.05 °C. Fibre optic probes with higher resolution would be desirable to reduce the tolerance. Also, Buchenberg et al. reported that certain types of fibre optic probe, including the type used in our experiments, can be sensitive to the B_0_ field strength causing changes in absolute temperature measurement (but not leading to errors in relative temperature measurement) [22].

## 5 Conclusions

We have presented a method for phantom characterisation and an MR thermometry technique for RF safety evaluation that provides full phantom coverage and increased sensitivity using two RF shim acquisition modes and four echo times. This approach provides good coverage of the phantom and enables characterisation of each individual channel. The simulated temperatures aligned well with the experimental values and the PRF measurements aligned well with those of the fibre optic probe to within 0.11 °C error between averaged fibre optic probe measurement and thermal estimation, and 0.16 °C for PRF thermometry and measured temperature from fibre optic probe at the edge of the phantom. Further studies may be needed to assess RF heating for the case of arbitrary pulse sequences and human models.

## Appendix A Supplementary data

Supplementary material Image 1
