# Supplementary material for: Assessment of radio-frequency heating of a parallel transmit coil in a phantom using multi-echo proton resonance frequency shift thermometry
Source: Magn Reson Imaging. 2021 Apr;77:57–68. doi: 10.1016/j.mri.2020.12.013 (PMC7889491; doi:10.1016/j.mri.2020.12.013)
Supplement: Supplementary file 1 — Supplementary material [file mmc1.docx]

**Supplemental online material:**

1. Phantom production procedure

The following procedure was adopted: firstly, DI water was boiled on a hot plate and mixed with sodium chloride (Sigma-Aldrich, St. Louis, MO) and benzoic acid (Fisher Scientific, Hampton, NH) using a magnetic stirrer. Agar powder (Sigma-Aldrich, St. Louis, MO) was mixed in until the colour of solution became transparent. The temperature of the hot plate was maintained below 300 C° to avoid burning the agarose with continuous stirring near the heat source. When bubbles started forming the heat source was turned off and TX-151 (Oil Research Company, United States) was used to decrease the setting time of solution, and sprinkled gradually while quickly stirring. The solution was filtered using a strainer to remove undissolved TX-151 and added into the phantom container along with the polyethylene powder (Sigma-Aldrich, St. Louis, MO) which is an insoluble ingredient and is mixed until it is evenly distributed. Any bubbles on the top were purged using a needle.

2. Dielectric properties measurement

To measure the dielectric properties of the phantom an N-type bulkhead connector was cut to have a flat surface with a round flange and central conductor and then soldered onto a semi-rigid coaxial cable (**Supplemental Figure 1**). An open and short calibration kit was made. For the ‘open’ probe a separate pair of identically cut N-type bulkhead connectors were connected facing each other; for the ‘short’ probe the signal and ground plate were connected with conductive material (**Supplemental Figure 2**). An N-type 50-ohm load was used for system calibration. Firstly the complex reflection coefficients were measured with the probe in de-ionised water [1]. Then the complex reflection coefficients were measured with the probe on the agarose phantom, and finally with the probe in saline water (3g NaCl per 1 L DI water) to verify the method. The conductivity and permittivity are determined using the real and imaginary reflection coefficients by solving a 5^th^ order polynomial equation as described in reference [2].


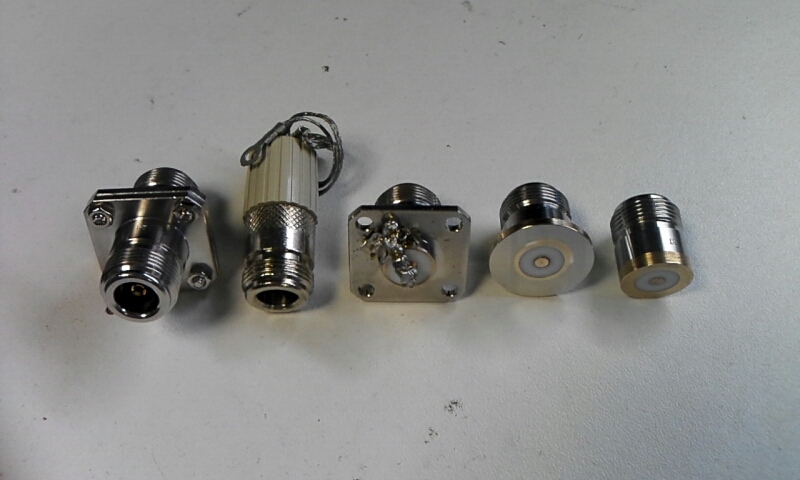


Supplemental Figure 1. The measurement probe


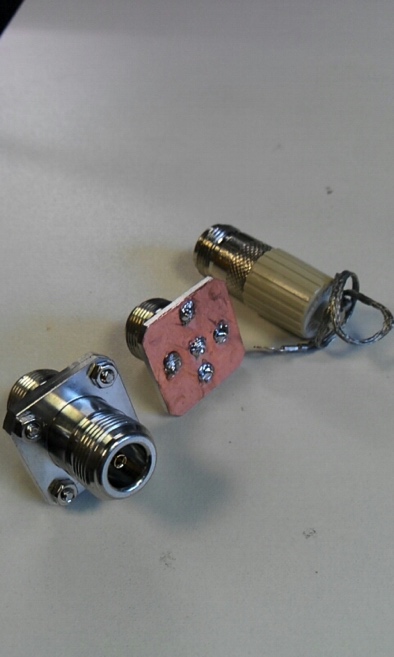


Supplemental Figure 2. Calibration standards

3. Potential source of artefact

Supplemental Figure 3 shows potential sources of artefact. An inadequately dissolved phantom ingredient positioned at the edge of the phantom caused the susceptibility artefact shown in the localisation image (Supplemental Figure 3a) resulting in a corresponding artefact shown in PRF thermometry (Supplemental Figure 3b). Also, the laminated print label produced a large artefact shown in Figure 10.

| 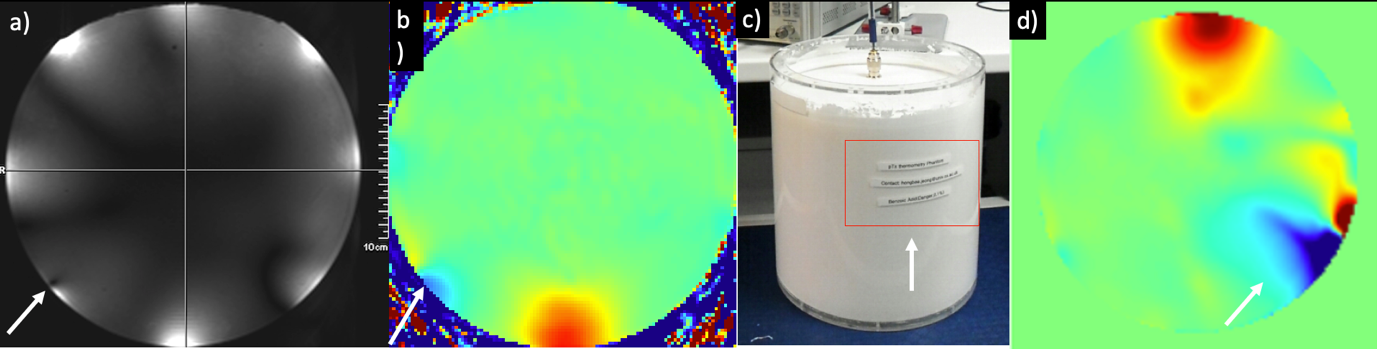 |
| --- |
| **Supplemental Figure 3**. **Potential sources of artefacts**. a) shows the location of the susceptibility artefact observed in the localisation image producing an artefact in the left-bottom region; b) shows the consequent non-heating related thermal artefact in the PRF reconstruction, c) shows the laminated labels attached outside the phantom as an institutional safety requirement, d) shows the large artefact caused by the laminated print label when it is included in field-of-view . |

4. Transmitted power calculation : MATLAB code

The following code was used to calculate the transmitted power generated by the fermi pulse (off-frequency additional heating), and the rectangular pulse (GRE imaging).

| prot=ascconvtrans(filename); % Open header file  [a b]=prot.sTXSPEC.aRFPULSE.flAmplitude  rect_v=str2num(a{1}); %27.7778 V  fermi_v=str2num(b{1}); %94.9241 V  %Averaged power from fermi pulse  TR = 0.014; % Repetition time  pulse_length_us = 900;  my_gradient = zeros(3,pulse_length_us/10); % gradient on 1µs raster time    % create firmi pulse for only one channel here  tstep = 1e-6;  t0 = (pulse_length_us - 500)*tstep/2;  a = tstep*10;  dW = 2*pi*20000; %10 khz off-frequency    t = ((1:10:pulse_length_us-500)*tstep - t0); %1400-500 = 900 500 for spoiling gradient  fermi = exp(1i*dW*t)./(1 + exp((abs(t) - t0)/a));    N_CH = 2;% Channel number using for heating – not relavant to heating  v_heating = fermi_v;  my_rf(N_CH,1:length(fermi)) = fermi*v_heating;  firmi_ave_energy_in_pulse = (sum(abs(my_rf(N_CH,:)).^2)*10e-6)/50;  fermi_ave_power = firmi_ave_energy_in_pulse/TR    % Averaged power from Rectangular pulse  pulse_length_us = 500;  my_gradient = zeros(3,pulse_length_us/10); % gradient on 10us raster time    my_rect = ones(N_CH,50)*rect_v; % Voltage for rectangular pulse  my_rf = zeros(N_CH,pulse_length_us/10);  my_rf(:,end-49:end) = my_rect;  Rect_ave_energy_in_pulse = (sum(abs(my_rf(N_CH,:)).^2)*10e-6)/50;  Rect_ave_power = Rect_ave_energy_in_pulse/TR    Delivered_P=fermi_ave_power+Rect_ave_power |
| --- |

5. TIAMO mode magnitude intensities

The magnitude images of two shim modes are displayed which shows the area of low signal intensities that could be improved with 2^nd^ shim mode.

| ** |
| --- |
| **Supplemental Figure 4**. **Magnitude intensities of two modes***, showing magnitude of signal in our experiments from our two transmit modes. These were acquired with (a) a 0° and (b) 45°* phase increment. |

Separately, signal intensity estimation was done using a Bloch simulation tool, SYSSIM (ZMT, Switzerland) [3], using the averaged brain properties for 7T (T1 relaxation time: 2137.5 ms, T2 relaxation time: 41.45 ms, Proton Density: 0.635) [4]. The sequence parameters were set equal to the experimental parameters used in 3D-GRE sequence for PRF thermometry (TR: 14 ms, TE: 9.26 ms, Flip Angle: 10˚), and the simulated B_1_^+^ and B_1_^-^ fields in CP mode and the zero-phase mode were used to estimate the signal intensity in 7T. Pennes’ bio-heat equation was used to estimate thermal elevation in CP-mode, zero-phase mode in 1258 s with 0.55 W of conducted power. The thermal simulation results of the CP-mode in single acquisition (41 s) is used as the initial thermal status of the 2^nd^ image mode (zero-phase) for 41 s. And the results of alternating image mode was used to estimate the thermal elevation in the TIAMO mode.

| 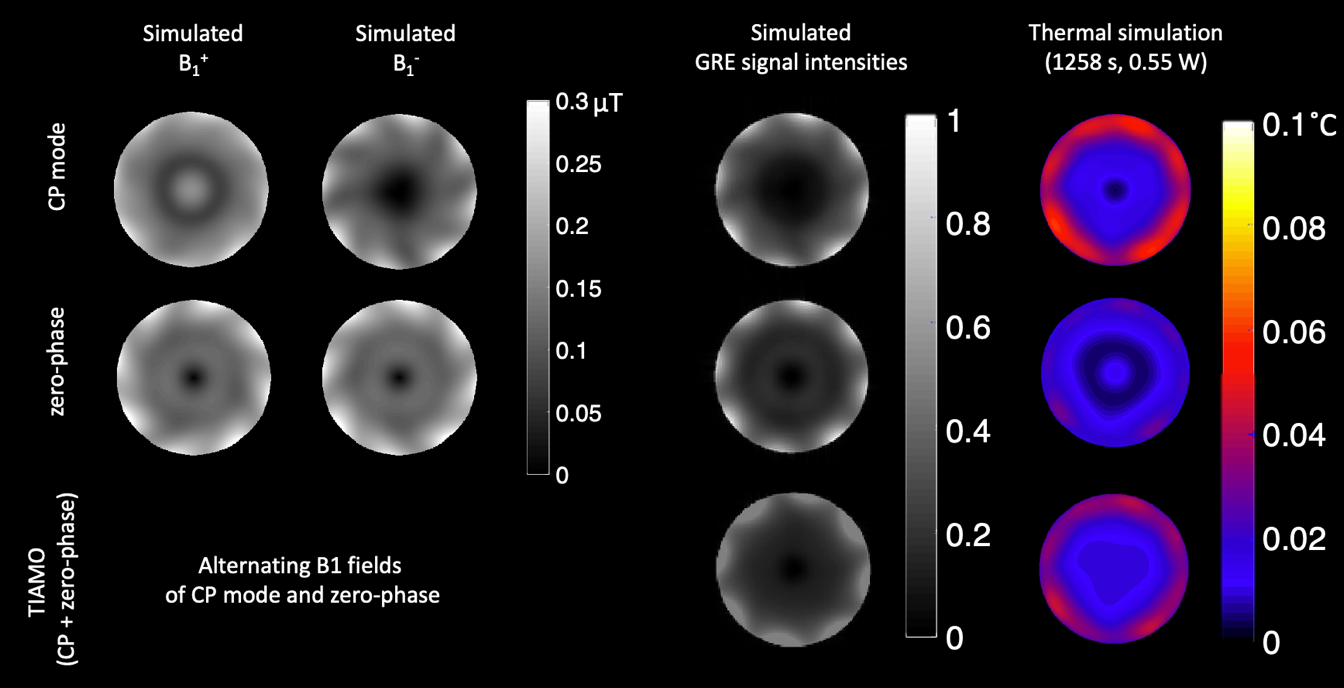 |
| --- |
| **Supplemental Figure 5**. **TIAMO mode analysis**. top) shows the simulation results of B_1_^+^ B_1_^-^fields, GRE signal intensities, and thermal simulation of 1258 s in CP-mode; middle) shows the simulation results of B_1_^+^, B_1_^-^ fields, GRE signal intensities, and thermal simulation of 1258 s in zero-phase mode; bottom) shows the GRE signal intensities, and thermal simulation of 1258 s in TIAMO mode . |

These results demonstrate the low receiver sensitivity in the centre of this relatively large phantom.

**References**

[1] Zajíček R, Oppl L, Vrba J. Broadband measurement of complex permitivity using reflection method and coaxial probes. Radioengineering 2008;17:14–9.

[2] Zajíček R, Vrba J, Novotný K. Evaluation of a Reflection Method on an Open-Ended Coaxial Line and its Use in Dielectric Measurements. Acta Polytech 2006;46:50–4. doi:10.14311/882.

[3] Cao Z, Oh S, Sica CT, McGarrity JM, Horan T, Luo W, et al. Bloch-based MRI system simulator considering realistic electromagnetic fields for calculation of signal, noise, and specific absorption rate. Magn Reson Med 2014;72:237–47. doi:10.1002/mrm.24907.

[4] Leroi L, Gras V, Boulant N, Ripart M, Poirion E, Santin MD, et al. Simultaneous proton density, T1, T2, and flip‐angle mapping of the brain at 7 T using multiparametric 3D SSFP imaging and parallel‐transmission universal pulses. Magn Reson Med 2020;84:3286–99. doi:10.1002/mrm.28391.
